# Phenotypic Characterization of Idiopathic Epilepsy in Border Collies

**DOI:** 10.3389/fvets.2022.880318

**Published:** 2022-05-12

**Authors:** Koen M. Santifort, Elise Bertijn, Sofie F. M. Bhatti, Peter Leegwater, Andrea Fischer, Paul J. J. Mandigers

**Affiliations:** ^1^Evidensia Small Animal Hospital, Arnhem, Netherlands; ^2^Department of Clinical Sciences, Utrecht University, Utrecht, Netherlands; ^3^Small Animal Department, Faculty of Veterinary Medicine, Ghent University, Merelbeke, Belgium; ^4^Centre for Clinical Veterinary Medicine, Clinic of Small Animal Medicine, Ludwig-Maximilians-University Munich, Munich, Germany

**Keywords:** canine epilepsy, dog, idiopathic epilepsy, seizure, hereditary

## Abstract

The prevalence of idiopathic epilepsy (IE) within the Border Collie (BC) dog breed is high. The aim of this retrospective study was to describe the phenotype of BCs with IE and assess correlations between phenotypic variables and owner-provided quality-of-life (QoL) scores. Data of BCs diagnosed with IE during the period of five consecutive years were retrospectively analyzed. All the dogs were presented at least once to a veterinary neurology specialist at one of three veterinary referral hospitals and most were under the continued medical care of that specialist. Owners were requested to complete a standardized online questionnaire including quality-of-life (QoL) scoring questions. Data of a total of 116 BC dogs were included for analysis. The median age at onset of the first epileptic seizure (ES) was 33.5 months (6–188). A total of 34/86 (40%) of medically treated dogs received 1 antiseizure medication (ASM) and 52/86 (60%) received ≥2 ASMs. Phenobarbital was the most commonly employed ASM, used in 70/86 of treated dogs (81%). Four or more side effects were observed in 20/86 (23%) of treated dogs. Age at onset of first ES was significantly lower for dogs having experienced cluster seizures (CSs), status epilepticus (SE), or both (median 27 months) vs. dogs that had not experienced CS or SE (median 43 months). The QoL of BC with IE was scored with a median score of 7 out of 10. Owners scored their dog's QoL to have declined by a median of 30% during the course of life with IE with 39% (37/95) of owners scoring their dog's QoL to have declined by ≥50%. This study confirms the association of age at onset of first ES with the severity of epilepsy (e.g., presence of CS and/or SE) and further characterizes the phenotype of IE in BC dogs. QoL of BC can be heavily impacted by IE.

## Introduction

The prevalence of epilepsy in the general dog population is estimated to be 0.6–0.75% (1–6). Breed-specific characteristics (phenotype or clinical manifestation) of idiopathic epilepsy (IE) have been reported for a variety of breeds, but most are based on single studies from specific countries with notable exceptions such as the Belgian Shepherd, Golden Retriever, and Finnish Spitz (6). Although it is well-established within the veterinary neurology community that Border Collie (BC) dogs show a high prevalence of IE (1–6), there is only one report that focuses on IE in this breed, based on a cohort of 49 BC dogs from Germany (7). Another study investigated IE in Labradors and Border Collies from the UK and the impact of neutering (8). Breed-specific differences regarding IE in dogs may hold important implications for owners, prognostication and successful clinical management (6). Observations of particular breeds with severe [exhibiting high-frequency epileptic seizures (ES), cluster seizures (CS), or status epilepticus (SE)] or antiseizure medication (ASM)-resistant IE in particular, may provide incentives for epilepsy research (e.g., genetic studies). Although a genetic basis was strongly indicated by observations from the earlier study on BC dogs (7) and clinical experience of many veterinary neurologists has suggested this for a long while, no causative genetic mutations or variations have been identified yet in the BC breed. The phenotype reported by Hülsmeyer et al. on a cohort of German BC dogs with IE was characterized by a high prevalence of CS occurring in almost all dogs (94%) and was further complicated by SE in about half of BCs (7). The presence of CS and/or SE is interpreted as IE being “severe” (4, 7). The main aim of this retrospective study was to evaluate and describe the phenotype of BCs with IE and assess correlations between phenotypic variables and owner-provided quality-of-life (QoL) scores.

## Materials and Methods

### Data Collection

Data were collected manually and retrospectively by reviewing digital patient files in the database of the Department of Small Animal Medicine, Faculty of Veterinary Medicine, Utrecht University, The Netherlands; Small Animal Department, Faculty of Veterinary Medicine, Ghent University, Merelbeke, Belgium and Evidensia Small Animal Hospital Arnhem, The Netherlands and review of data collected *via* an online questionnaire (see later). All the dogs were privately owned and participating owners gave informed consent for participation in the study. The dogs entered in this study were examined and handled by licensed veterinarians in compliance with local legislation and approval of an ethics committee for the study was not necessary. BC dogs were included if dogs had been presented for clinical consultation (including a general and neurological examination) with a specialist in veterinary neurology at one of three veterinary referral hospitals during a period of five consecutive years (2016–2021) and diagnostic test results were consistent with a diagnosis of IE [minimum tier I, International Veterinary Epilepsy Task Force (9)]. Most dogs were under continued care by that specialist. All the BCs had to have a pedigree or descended directly from BCs with a pedigree. Cases were excluded when they did not fit the inclusion criteria noted earlier or when data were insufficient. Insufficient data were defined as data for a particular dog that did not include parameters noted below marked with “^*^” or ≥3 of the remaining parameters under investigation were not recorded in the files. Data recorded consisted of: breed (Border Collie)^*^; sex^*^; neuter status^*^; alive (yes/no)^*^; age at death (when dead)^*^; age at the time of data collection^*^; age at onset of first ES (first ES noticed by owner)^*^; ASM treatment (yes/no)^*^; ASM or ASMs used^*^; occurrence of side effects (yes/no); type of side effects; type of ES (generalized, focal or combination); type of focal ES (motor, autonomic, behavioral); ES frequency in the last 3 months; occurrence of CS (yes/no); occurrence of SE (yes/no); owner-identified triggers or temporal pattern (yes/no); type of trigger or temporal pattern; occurrence of pre-ictal signs (yes/no); type of pre-ictal signs (yes/no); occurrence of post-ictal signs (yes/no); type of post-ictal signs (yes/no); vaccination status up-to-date (yes/no); anthelmintic drug use (yes/no); and anti-ectoparasitic drug use (yes/no; flea/tick prevention). Data were transferred to Microsoft® Excel (2021) for analysis. Cluster seizure and SE were defined as: CS = two or more ES within a 24-h period, SE = >5 min of continuous ES or two or more discrete ES between which there is incomplete recovery of consciousness (for generalized convulsive ES) (10). ES pertaining to 1 CS event were counted as 1 ES for ES frequency.

### Quality-of-Life

Owners of included BCs were requested to complete a standardized online questionnaire including quality-of-life (QoL) scoring questions adapted from other studies (11) at variable times during their dog's clinical course. Owners were asked to provide further detailed information on the dog (used to confirm data stored in patient files, such as date of birth and yes/no answer to questions already noted during consultations) and their personal information (e.g., name, e-mail address and phone number; voluntarily provided with owners' permission, used in case answers to the questions needed further explanation). Owners were asked to score the QoL of their dog with IE on a scale of 1 (very bad)-10 (very good), and also to score the QoL of their dog with IE as a percentage compared to the QoL before the onset of IE (baseline score of 100%). The original questionnaire was in Dutch. Questions were translated into German and English for owners not able to complete the Dutch questionnaire.

### Statistical Analysis

Descriptive statistics are reported, a chi-square or the Mann–Whitney *U*-test (2-tailed) was performed to test for significant differences (*P* < 0.01 was considered significant) between subgroups of the study population for categorical and continuous data, respectively, and a McNemar test was used to evaluate the association between clinical variables within a group (paired, nominal data). Pearson correlation tests [reported as r(df) = r, P] were performed to evaluate and describe if there were significant relationships (*P* < 0.01 were considered significant) between clinical variables using Microsoft® Excel (2021). Correlations (r) were interpreted as: 0.90–1.00 (−0.90– −1.00) = very strong to perfect positive (negative) correlation; 0.70–0.90 (−0.70– −0.90) = strong positive (negative) correlation; 0.50–0.70 (−0.50– −0.70) = moderate positive (negative) correlation; 0.30–0.50 (−0.30– −0.50) = weak positive (negative) correlation; 0.00–0.30 (−0.00– −0.30) = no to very weak positive (negative) correlation. Odds ratios were calculated and reported with 95% CIs. All the other results are presented as medians and ranges: median (range lowest–highest) unless stated otherwise.

## Results

A total of 185 BC dogs with IE were identified in the databases of included institutions. A total of 69 cases were excluded because did not meet inclusion criteria. A total of 116 BC dogs met the inclusion criteria, had sufficient data recorded, and were included for analysis. In total, 78 of the included dogs originated from The Netherlands, and 33 from neighboring countries (Germany, Belgium), and 5 dogs were of undefined origin. Study population characteristics are summarized in Table 1.

**Table 1:** Study population characteristics.

			**Number**	**Percentage**	
Total no. of dogs			116	100%	
Sex					
	Male	Total	66	57%	100%
		Intact	38		58%
		Neutered	28		42%
		Alive	52		79%
		Dead	14		21%
	Female	Total	50	43%	100%
		Intact	26		52%
		Neutered	24		48%
		Alive	36		72%
		Dead	14		28%
Neuter status		Intact	64	55%	
		Neutered	52	45%	
Alive			88	76%	
Dead			28	24%	

### Age at the Time of Data Collection, Age at Onset, Age at Death

The median age of all included dogs, either age at the time of data collection or age at the moment of death, was 45 months (6–188). Of those BCs still alive at data collection (*n* = 88), the median age at the time of data collection was 48 months (9–166). Of those BCs that had died (*n* = 28), the median age at death was 33.5 months (6–188). Causes of death were not recorded for any of the dead patients. The median age at onset of first ES was 33.5 months (6–174); there were no significant differences in age at onset of first ES between dogs that were dead or alive. No sex predisposition or association with neuter status was found regarding ES frequency, age at onset of first ES or mortality.

### ASM Treatment, ASMs Used, and Side Effects of Treatment

A total of 86/116 dogs (74%) were treated with ASM, 30/116 dogs (26%) were not treated with ASM. There were no differences in age at the time of data collection or age at onset of first ES between dogs treated or untreated. There were no statistically significant differences between male and female dogs, neutered and intact dogs, and alive or dead dogs with respect to being treated or not. A total of 34/86 (40%) of treated dogs were treated with 1 ASM and 52/86 (60%) were treated with ≥2 ASMs. Phenobarbital was the most commonly employed ASM (70/86, 81%), followed by potassium bromide (39/86, 45%), imepitoin (26/86, 30%), diazepam (18/86, 21%), levetiracetam (12/86, 14%), and gabapentin (2/86, 2%). The combination of phenobarbital/potassium bromide was the most common polytherapy employed (31/86, 36%).

The occurrence of side effects was reported in 70/86 (81%) of dogs treated with ASMs based on owner reports and clinical assessments. No significant associations were found between specific ASMs and the occurrence of side effects (yes/no) or the number of ASMs used in the treatment and the number of side effects. The most common side effect reported was lethargy (53%). Side effects are summarized in Table 2.

**Table 2:** Side effects of antiseizure medication (ASM) treatment.

	**Number of dogs (*n*)**	**%**
Dogs treated with ASM	86	100%
Yes (side effects)	70	81%
No (no side effects)	16	19%
**Type of side effect (n** **=** **70 dogs experiencing side effects)**		
Lethargy	43	61%
Polyphagia	31	44%
Ataxia	30	43%
Polydipsia	24	34%
Weakness	23	33%
Weight gain	20	29%
Polyuria	19	27%
Restlessness	13	19%
Defecation in house	3	4%
Diarrhea	2	3%
Skin rash	1	1%
Coughing	1	1%
**Number of side effects (n** **=** **86 dogs treated with ASM)**		
No side effects	16	19%
1 side effect	14	16%
2 side effects	15	17%
3 side effects	20	23%
≥4 side effects	21	24%

*There were no statistically significant associations between types of side effects and ASM used*.

### Frequency of ES, Type of ES, and Occurrence of CS and SE

The ES frequency during the last 3 months was reported in 76 dogs. The median ES frequency per month was 1 (0–17). There was no significant difference in ES frequency between dogs <24 months of age at onset of first ES vs. dogs ≥24 months of age at onset of first ES. No significant correlation was found between age at onset of first ES or age at the time of data collection and ES frequency. Dogs that were treated with ASMs did not have a significantly higher ES frequency than untreated dogs in the previous 3 months.

The occurrence of CS and SE was reported in 114 dogs (Table 3). Dogs reported having experienced SE were significantly more likely to have experienced CS than not (and *vice versa*). Age at onset of first ES was significantly lower for dogs having experienced CS, SE or both [27 months (4–165)] vs. dogs that had not experienced CS or SE [43 months (5–174)]. Dogs having experienced CS, SE, or both were more likely to be dead than dogs not having experienced CS or SE with an odds ratio of 3.33 (1.54–8.00, 95% CI). There was no significant association between dogs being treated or not and being treated with 1 or >1 ASMs vs. having experienced CS, SE, or both.

**Table 3:** Number and percentages of dogs experiencing cluster seizures (CS) and status epilepticus (SE) (*n* = 114).

	**CS**		
	**Yes**	**No**	**Total**
**SE**			
Yes	25 (22%)	8 (7%)	33 (29%)
No	42 (37%)	39 (34%)	81 (71%)
Total	67 (59%)	47 (41%)	114 (100%)

The most common type of ES reported was generalized tonic–clonic (102/116, 88%) with only 8/116 (7%) of dogs experiencing focal ES and 5/116 (4%) experiencing a combination of both types. Out of 8 dogs experiencing focal ES, 4 were reported to present as motor dysfunction, 3 were reported to present as autonomic dysfunction and 1 was reported to present as behavioral dysfunction.

### Triggers, Temporal Pattern, Pre-Ictal Signs, and Post-Ictal Signs

The occurrence of triggers or temporal patterns for ES was reported in 114 dogs (Table 4). In 68/114 (60%) dogs, no triggers or temporal patterns were identified. In the 46/114 (40%) dogs where triggers or temporal patterns were identified, stress was reported as a trigger in 22/46 (48%).

**Table 4:** Triggers or temporal patterns identified by owners.

	**Number of dogs (*n*)**	**%**
**Trigger or temporal pattern (*****n*** **=** **114 dogs for which these were or were not reported)**		
Yes (identified)	46	40%
No (not identified)	68	60%
**Type of trigger or temporal pattern (*****n*** **=** **46 dogs for which these were reported)**		
Stress	22	48%
Time of day (morning, evening or night)	17	37%
Influence of weather	7	15%
Physical exercise	6	13%
Sexual excitement	4	9%
Visit veterinarian	4	9%
Seasonal influence	1	2%
Other	2	4%

The occurrence of pre- and post-ictal signs was reported in 115 dogs (Table 5). In 64/115 (56%) dogs, pre-ictal signs were reported. Seeking contact with the owner (29/64, 45%) and restlessness (26/64, 41%) were the most frequently reported pre-ictal signs. In 95/115 (83%) dogs, post-ictal signs were reported. Walking aimlessly (79/95, 83%) and lethargy (60/95, 63%) were the most frequently reported post-ictal signs.

**Table 5:** Pre- and post-ictal signs.

	**Number of dogs (*n*)**	**%**
Pre-ictal signs	115	100%
Yes (identified)	64	56%
No (not identified)	51	44%
Post-ictal signs	115	100%
Yes (identified)	95	83%
No (not identified)	20	17%
Type of pre-ictal signs	64	
Seeking contact with owner	29	45%
Restlessness	26	41%
Salivation	4	6%
Vomiting	2	3%
Aggression	2	3%
Other	5	8%
Type of post-ictal signs	95	
Walking aimlessly	79	83%
Lethargy	60	63%
Drinking	28	29%
Blindness	25	26%
Eating	24	25%
Aggression	7	7%
Other	13	14%

### Vaccination Status, Anthelmintic Drug Use, and Anti-Ectoparasitic Drug Use

The vaccination status was reported for 104 dogs and was up-to-date in 82 (79%) of dogs. For 103 dogs, the application of anthelmintic and anti-ectoparasitic was reported. Anthelmintic drugs were used in 72/103 (70%) dogs and anti-ectoparasitic were used in 66/103 (64%) dogs. No associations were found between these variables and age at onset of first ES, age at the time of data collection, the occurrence of CS, SE, or both, ES type, or ES frequency.

### Quality-of-Life

The QoL of BC with IE was scored by 95 out of 116 (82%) owners with a median score of 7 (1–10). Dogs having experienced CS, SE, or both were scored significantly lower QoL scores [7 (1–10)] by their owners than dogs that had not experienced CS, SE, or both 9 (1–10) (*P* = < 0.01). Dead dogs (scored QoL pertaining to when they were alive) were scored a median of 2 (1–10), which was statistically significantly different from dogs that were alive (median score of 8, 3–10) (*P* = < 0.01). QoL scores were not significantly impacted by treatment with ASM(s) (yes/no) or the occurrence of side effects (yes/no) and were not significantly associated with age at the time of data collection, age at onset of first ES or ES frequency.

Owners scored their dog's QoL to have declined by a median of 30% during the course of life with IE with 39% (37/95) of owners scoring their dog's QoL to have declined by ≥50%. The owners' perception of change in QoL showed a very weak positive correlation with age at the time of data collection [i.e., the older the dog was, the lower the change in QoL (less negative impact)] [r (93)= 0.30, *P* = < 0.01]. Dogs that were dead (QoL pertaining to when the dog was still alive) were scored to have 90% (0–100) reduction in QoL, which was a statistically significantly higher change compared with a reduction of 30% (0–80%) for those dogs that were still alive (*P* = < 0.01). The amount of change in QoL was not significantly impacted by treatment with ASM(s) (yes/no) or the occurrence of side effects (yes/no) and was not significantly associated with age at onset of first ES or the occurrence of CS, SE, or both.

## Discussion

This study provides additional information characterizing the phenotype of IE in BC dogs. Several findings reported previously were mirrored in our study that included a larger number of BC dogs (7). The median age at onset of first ES was 33.5 months (2.8 years) compared with 2.4 years in the study by Hülsmeyer et al. (7). There was no sex predisposition or association with neuter status. The most frequently recorded owner-reported trigger was unspecified stress. Restlessness and seeking owners' attention were recorded as the most prevalent pre-ictal signs. The predominant ES type was a generalized tonic-clonic ES. Only a few dogs had (additional) focal ES that were characterized as either autonomic, behavioral or motor. Walking aimlessly and lethargy were the most common identified post-ictal signs. Although restlessness was reported as the most common post-ictal sign in the earlier study, “walking aimlessly” may be described as “restlessness”. This elucidates the difficulty in the interpretation of signs reported by owners and their subjective nature. In total, 74% of dogs were treated with ASMs, compared with 78% of dogs in the study by Hülsmeyer et al. (7). However, as only 33 treated dogs were included for further analysis in that study, our subset of 86 dogs provided further information on which ASMs were mostly employed (phenobarbital in 81%) and how many were used to treat these dogs. In total 60% of BC treated with ASMs were treated with 2 or more ASMs. While we did not evaluate response to treatment or ASM resistance, this finding is speculatively suggestive of a difficult to treat form of IE, in accordance with the study by Hülsmeyer et al. (7). In the same study, ASM side effects were reported by 67% of owners of BCs with IE, vs. 81% of owners of BCs with IE in our study. In almost a quarter of these dogs, 4 or more side effects were reported. These findings highlight the importance of discussing the possibility of the occurrence of side effects with owners of BCs with IE when treatment is contemplated or started. Most reported side effects are well-known to be associated with the use of ASMs in the treatment of dogs with IE (12). An important difference in findings between our study and that of Hülsmeyer et al. is the prevalence of SE and CS in BC with IE (7). CS occurred in 94% of dogs in that study vs. 59% in our study and SE occurred in 53 vs. 29%, respectively. These differences may be explained, for instance, by differences in treatment, collection bias (differences in methods and the possibility that owners of severely affected dogs are more likely to respond to questionnaire calls), dependence on owner reports, and differences in the severity of IE in the cohorts studied. Still, a significant number of dogs in our study experienced CS and SE. The occurrence of CS and/or SE in various dog breeds with IE has been reported and is highly variable, but consistently high in BCs and notably higher than in some other breeds, such as the poodle or Finnish Spitz (13–18).

We chose to include an owner-reported ES frequency over the last 3 months as owner-recall bias would be expected to have a larger influence on ES frequency calculated over longer periods of time than shorter periods of time. The time frame of ES frequency calculated over the last 3 months has been applied in the recent studies and we chose this time frame as most owners had ES diaries pertaining to that period (19). Still, as the moment of data collection was not standardized (e.g., before starting ASM or after, the time elapsed since age at onset of first ES was variable et cetera), the interpretation and analysis of these data are confounded. This should be taken into account when interpreting findings relating to ES frequency and associations with other variables. No significant associations between ES frequency and other clinical variables were found in this study.

The finding that age at onset of first ES was significantly lower for dogs having experienced CS, SE, or both and that these dogs were more likely to be dead than dogs not having experienced CS or SE is noteworthy and implies a worse prognosis. QoL scores for dead dogs were significantly low and QoL score changes were significantly higher than that for dogs that were alive. Although the authors would speculate that it is likely that euthanasia or epilepsy-related death was the reason for death in most of these cases, the reason for death was not recorded in this study and so this remains unclear. The occurrence of probable sudden unexpected death in dogs with epilepsy (pSUDED) has been studied recently and researchers found it likely that dogs with CS have an increased risk of pSUDED (20). We did not find any associations between the implementation of vaccinations or the use of anthelmintic or anti-ectoparasitic drugs and clinical variables and ES frequency or the occurrence of CS/SE or both. However, our data did not include information on the types of drugs used. This finding may provide dog owners in general and also those of dogs with IE with more evidence-based information to make their decisions on whether or not to vaccinate their dog with IE.

To evaluate the QoL of BC with IE, we asked owners to score the QoL of their dog with IE and separately also score the QoL of their dog as a percentage compared with the QoL before the onset of ES. The median QoL score was 7 out of 10. This may be subjectively interpreted as good despite the seriousness of IE in BCs. QoL scores were not found to be associated with treatment with ASMs. This may be explained by either a type II error (i.e., too small sample) or a multitude of conflicting effects of treatment (i.e., improvement of ES frequency or severity vs. the occurrence and severity of side effects). We did find a significant association between QoL scores of dogs with or without CS and/or SE: dogs with CS and/or SE had a significantly lower score than dogs without CS and/or SE. This finding implies that increased severity of IE leads to a decreased owner-scored QoL. This seems very logical and the presence of CS and/or SE has indeed been interpreted in the literature as an indicator of the severity of epilepsy (4, 7). More detailed studies on the QoL of BC with IE based on more exhaustive questionnaires may provide more insights on this matter (21). In total, thirty-nine percent (39%) of owners scored their dogs' QoL to have declined by ≥50%, which exemplifies the impact IE can have on the QoL of dogs. The very weak positive correlation of QOL with age at the time of data collection should not be overinterpreted. It is very weak to start with and may simply reflect, for instance, that dogs of older age at the time of data collection are less heavily impacted by side effects of treatment (e.g., lethargy may be less noticeable in a 10-year-old BC than in a 1-year-old BC) or less severe course of IE in elderly BCs.

The prevalence of IE in the BC breed has been shown to be high in many studies (1–6). Although a strong indication for a genetic background to the occurrence of IE in BC dogs is reported based on pedigree analysis (7), the causal genetic mutation(s) have not been identified yet. This holds true for many other dog breeds with a reported high prevalence of IE (6). It should be noted that a higher than average prevalence (or incidence) has been reported in some Dutch dog breeds specifically (e.g., Friesian Stabijhoun and Drentse Patrijshond) (6). A geographical predisposition for IE in BC (i.e., the Dutch BC) may be investigated by comparing incidence or prevalence between different geographical regions or countries. This would be best implemented by comparing the diagnostic percentage of national databases for IE in the BC (e.g., UK “VetCompass^TM^” and the Dutch “Petscan”) (5). If national databases become widely implemented, a geographical “heat map” of high incidences of IE could be constructed that may help select subpopulations of BC to investigate further. As the genetic basis for IE is likely complex and cooperation of kennel clubs, owners, and veterinarians is necessary to acquire large volumes and adequate samples for genetic analysis, this may provide helpful insights. Also, up-to-date information on the incidence of IE in the BC is very helpful in evaluating measures taken by breeders to reduce the prevalence of IE within the breed.

There are some notable limitations to our study. Due to its retrospective nature, the diagnostic work-up varied within the study population. By including dogs that fit the criteria for a tier I International Veterinary Epilepsy Task Force diagnosis of IE, we aimed to exclude to a degree dogs with structural causes of epilepsy or reactive seizures (9). However, we cannot exclude the possibility that some of the included dogs did not have IE. In addition, some of the recorded data were unspecific. For instance, by only noting the type of ES as “generalized tonic–clonic”, “focal” or “combination”, we were unable to discriminate between dogs having focal ES evolving to become generalized and those that had immediately generalized ES with certainty (6, 7, 10). Indeed, ES with the focal onset and secondary generalization were indicated in BC in the study by Hülsmeyer et al. but the definition thereof remains open to discussion (4). Other limitations are, among others, that the data did not allow for evaluation of the response to treatment or drug resistance and the subjective interpretation of some of the parameters by owners. Despite these limitations, this study provides valuable insights into the phenotype of BC with IE.

In conclusion, this study confirms the association of age at onset of first ES with the severity of IE (e.g., presence of CS and/or SE) and provides additional information characterizing the phenotype of IE in BC dogs. Age at onset of first ES was significantly lower for dogs having experienced CS, SE, or both. QoL of BC can be heavily impacted by IE and especially in BCs with CS.

## Data Availability Statement

The raw data supporting the conclusions of this article will be made available by the authors, without undue reservation.

## Author Contributions

PM was responsible for the study conception and data collection in cooperation with co-authors and supervised data analysis and manuscript editing, in cooperation with EB, SB, PL, and AF. The majority of Dutch cases were seen by PM and the majority of Belgium cases by SB. Statistical analysis, data analysis, and manuscript writing were performed by KS. All authors contributed to the article and approved the submitted version.

## Funding

This manuscript was financially supported by the University of Utrecht, through Open Access Publishing Funding.

## Conflict of Interest

The authors declare that the research was conducted in the absence of any commercial or financial relationships that could be construed as a potential conflict of interest.

## Publisher's Note

All claims expressed in this article are solely those of the authors and do not necessarily represent those of their affiliated organizations, or those of the publisher, the editors and the reviewers. Any product that may be evaluated in this article, or claim that may be made by its manufacturer, is not guaranteed or endorsed by the publisher.
