# Supplementary material for: Phenotypic Characterization of Idiopathic Epilepsy in Border Collies
Source: Front Vet Sci. 2022 May 12;9:880318. doi: 10.3389/fvets.2022.880318 (PMC9133619; doi:10.3389/fvets.2022.880318)
Supplement: Supplementary file 1 [file Data_Sheet_1.PDF]

Informatie over uzelf. Invoeren is niet verplicht.

1. Wat is uw naam? (invoeren is niet verplicht)

2. Adres? (invoeren is niet verplicht)

3. Postcode? (Invoeren is niet verplicht)

4. Woonplaats? (Invoeren is niet verplicht)

5. Wat is uw emailadres? (Invoeren is niet verplicht maar wel gewenst zodat we u bij onduidelijkheden achteraf kunnen bereiken).

6. Wat is uw telefoonnummer? (Invoeren is niet verplicht)

Vragen over uw hond, vaccinatie etc voorgeschiedenis.

7. Roepnaam van uw hond?

8. Stamboeknaam (indien van toepassing):

9. Stamboomnummer (graag volledig nummer inclusief cijfers van de uitgever. Dus bijvoorbeeld NHSB 58410000). Indien de hond geen stamboom heeft vermeld dan NVT.

10. Chipnummer van uw hond?

11. Wat is de geboortedatum van uw hond?

Datum

Datum

DD/MM/JJJJ

12. Wat is het geslacht van uw hond?

- Mannelijk
- Vrouwelijk

13. Is uw hond gesteriliseerd of gecastreerd?

- Ja
- Nee

14. Indien uw hond is gesteriliseerd: weet u nog bij benadering de datum waarop dit plaats heeft gevonden?

Datum/tijd

Datum

MM/DD/JJJJ

15. U heeft een Border Collie. Geef aan wat voor u van toepassing is. Meerdere antwoorden zijn mogelijk.

- [ ] Raszuiver (met stamboom)
- [ ] Raszuiver (zonder stamboom)
- [ ] Kruising of ander ras. Indien het een kruising is geef dan hier aan met welke ander ras. Indien het om een raszuiver ander ras gaat vul hier dan het ras in:

16. Werd uw hond gevaccineerd voordat hij/zij de eerste epileptiforme aanval kreeg?

- [ ] Ja
- [ ] Nee
- [ ] Indien het antwoord JA is. Welk merk? Dit kunt u in het vaccinatieboekje terug vinden.

17. Kreeg uw hond ontwormingstabletten voordat hij/zij de eerste epileptiforme aanval kreeg?

- [ ] Ja
- [ ] Nee
- [ ] Indien JA: weet u ook wat het merk van deze ontworming was?

18. Heeft u uw hond behandeld met anti-vlooien/teken producten voordat hij/zij de eerste epileptiforme aanval kreeg?

- [ ] Ja
- [ ] Nee
- [ ] Indien JA: weet u ook van welk merk dit product was?

19. Heeft uw hond een medicatie (anders dan hierboven staat) gekregen voordat hij/zij de eerste epileptiforme aanval kreeg?

- [ ] Ja
- [ ] Nee
- [ ] Indien Ja: welke medicatie was dit?

20. Wat was de voeding die uw hond kreeg voordat hij/zij de eerste epileptiforme aanval kreeg (Indien mogelijk merk vermelden)?

Type voeding 1

Type voeding 2

Type voeding 3

21. Is de voeding die hij/zij nu krijgt veranderd?

- ◯ Ja
- ◯ Nee

22. Wat voor soort voeding geeft u nu? (Indien mogelijk merk vermelden)

Type voeding 1

Type voeding 2

Type voeding 3

23. Heeft uw hond, voordat hij/zij de eerste epileptiforme aanval kreeg, een serieuze ziekte gehad? Indien u twijfelt vul dan altijd Ja in en vermeld welke ziekte het was.

- ☐ Ja
- ☐ Nee
- ☐ Indien JA: vul dan hier in wat voor een ziekte/ziektes het was.

Algemene vragen over uw woonsituatie en uw hond.

24. Hoe woont u? (meerdere antwoorden zijn mogelijk)

- [ ] Stad
- [ ] Dorp
- [ ] In een bosrijke omgeving
- [ ] Landelijk
- [ ] Aan zee
- [ ] Overige (geef nadere toelichting)

25. Heeft u nog andere huisdieren?

- ○ Ja
- ○ Nee
- ○ Indien ja:

26. Hoe omschrijft u het karakter van uw hond? (meerdere antwoorden zijn mogelijk)

- [ ] Levendig
- [ ] Opgewekt
- [ ] Kalm
- [ ] Zenuwachtig
- [ ] Angstig
- [ ] Agressief
- [ ] Overige (geef nadere toelichting)

27. Leeft uw hond grotendeels binnen of buitenhuis?

- [ ] Binnenhuis
- [ ] Buitenhuis
- [ ] Een combinatie van beide
- [ ] Overige (geef nadere toelichting)

28. Hoe veel uren per dag is uw hond onder uw, of van een van uw huisgenoten, toezicht?

- ○ Minder dan 5 uur per dag
- ○ Vijf tot 10 uur per dag
- ○ Tien tot 15 uur per dag
- ○ Vijftien tot 20 uur per dag
- ○ Meer dan 20 uur per dag

Vragen over de (eerste) aanvallen.

29. Wanneer heeft u de eerste aanval bij uw hond gezien? Indien u de datum niet exact meer weet, geef dan een datum bij benadering.

Datum

Datum

DD/MM/JJJJ

30. Wanneer is de meest recente aanval opgetreden?

Datum/tijd

Datum

DD/MM/JJJJ

31. Heeft uw hond meer dan 1 aanval gehad?

- Ja
- Nee

32. Hoeveel aanvallen heeft uw hond tot op heden gehad?

33. Weet u wat de oorzaak van de aanvallen bij uw hond is? Slechts een antwoord is mogelijk

- Bewezen genetisch
- Idiopathisch (dit wil zeggen: we weten de oorzaak niet, vaak vermoedelijk genetisch)
- Metabool / toxisch / reactief (= de oorzaak ligt buiten de hersenen. Denk aan bijv een lever, nier of suikertekort)
- Structureel / symptomatisch / secundair (= de oorzaak zit in de hersenen. Denk aan bijv trauma, ontsteking, tumor)
- Overige (geef nadere toelichting)

34. Wie heeft deze diagnose gesteld?

- [ ] Ikzelf
- [ ] Mijn dierenarts
- [ ] De specialist
- [ ] Overige (geef nadere toelichting)

35. Wat voor type aanval vertoont uw hond vooral?

- ○ Gegeneraliseerde tonisch-clonische aanvallen. Dit zijn aanvallen waarbij de hond in de regel op zijn zijde ligt, krampen en fietsbewegingen heeft, trekkingen met de mond, in de regel krijgt u geen contact met hem/haar en kan speeksel, urine en ontlastingsverlies optreden.
- ○ Focale of partiele aanvallen. Dit zijn, bijvoorbeeld, aanvallen waarbij slechts een deel van het lichaam de abnormaliteit laat zien. Voorbeelden zijn: trekken met de lippen, speekselen, alleen een pootje etc.
- ○ Een combinatie van zowel tonisch-clonische aanvallen en de focale aanvallen.
- ○ Overige, namelijk...

36. Wie is de behandelaar van de epilepsie bij uw hond?

- ○ (Eerstelijns) dierenarts
- ○ Specialist veterinaire neurologie
- ○ Specialist veterinaire neurologie in opleiding (SIO)
- ○ Specialist interne geneeskunde
- ○ Overige (geef nadere toelichting)

37. Hoe bent u terecht gekomen bij de specialist?

- ○ Dit heb ik zelf geregeld
- ○ Dit heb ik gedaan op advies van mijn dierenarts
- ○ Niet van toepassing
- ○ Anders, namelijk...

38. Is uw hond nog in leven?

- Ja
- Nee

39. Indien uw hond overleden is: op welke leeftijd is uw hond overleden?

40. Wat was de oorzaak van het overlijden van uw hond?

- Epilepsie
- Anders, namelijk...

41. Hoeveel aanvallen had uw hond in het EERSTE jaar nadat de eerste aanval is gezien?

Aantal aanvallen per jaar

Aantal aanvallen de eerste zes maanden

Aantal aanvallen de eerste drie maanden

Aantal aanvallen de eerste maand (indien van toepassing)

Aantal aanvallen de eerste week (indien van toepassing)

42. Hoeveel aanvallen heeft uw hond dit LAATSTE jaar gehad?

Niet van toepassing: dit is pas het eerste jaar

Aantal aanvallen het laatste jaar

Aantal aanvallen de laatste zes maanden

Aantal aanvallen de laatste drie maanden

Aantal aanvallen de laatste maand (indien van toepassing)

Aantal aanvallen de laatste week (indien van toepassing)

43. Hoe beoordeelt u de ernst van de aanvallen NU ten opzichte van de eerste aanvallen. U kunt nu met de schuifbalk aangeven of het minder of erger is geworden. Indien u de balk in het midden zet is het gelijk gebleven. Naar links minder en naar rechts meer. Nul = niet ernstig, 10 is heel ernstig.

0 10

44. Hoe lang heeft het geduurd voordat u met een anti-epilepticum bent gestart: gerekend vanaf de eerste aanval die u heeft gezien.

- ◯ We zijn gelijk, de eerste week, na de eerste aanval begonnen met een medicatie.
- ◯ We zijn nog niet begonnen met een medicatie.
- ◯ We hebben gewacht: vul hier het aantal maanden tussen de eerste aanval en de start van de medicatie in.

45. Kunt u een trigger (aanleiding) aanwijzen voor het optreden van de aanvallen? Meerdere antwoorden zijn mogelijk.

- ☐ Stress
- ☐ Sexuele opwinding
- ☐ Weersinvloeden
- ☐ Bezoek aan huis
- ☐ Bezoek aan de dierenarts
- ☐ Er is een duidelijk tijdstip van de dag aanwezig
- ☐ Er is een duidelijke seizoensinvloed aanwezig
- ☐ Ik herken geen factoren die de aanvallen triggeren
- ☐ Geef hier een nadere toelichting op het bovenstaande antwoord.

46. Indien uw hond gesteriliseerd of gecastreerd is. Is dit uitgevoerd NADAT de hond aanvallen heeft ontwikkeld?

- ◯ Ja
- ◯ Nee
- ◯ Niet van toepassing

47. Indien uw hond is gesteriliseerd of gecastreerd nadat hij/zij de aanvallen heeft ontwikkeld. Wat is dan voor u van toepassing?

- ◯ De aanvallen namen in frequentie af.
- ◯ De aanvalsfrequentie veranderde niet.
- ◯ De aanvalsfrequentie nam toe
- ◯ Overige (geef nadere toelichting)

48. Is uw hond verwant aan andere epilepsielijders?

- ◯ Ja
- ◯ Nee
- ◯ Weet ik niet
- ◯ Indien ja wat is de verwantschap

Aanvullende vragen over de aanvallen.

49. Vaak zien we een inleidende fase. Dat is niet de aanval zelf maar een fase (seconden tot dagen voor de werkelijke aanval) voor de aanval. Herkent u deze bij uw hond?

- Ja
- Nee
- Weet ik niet

50. Indien u een inleidende fase ziet: kunt u deze dan beschrijven?

- Misselijkheid
- Braken
- Speekselen / kwijlen
- Rusteloosheid
- Hij/zij zoekt contact met mij/ons
- De hond wordt agressief
- Overige (geef nadere toelichting)

51. Indien er een inleidende fase is: hoe lang voordat de aanval optreedt ziet u deze fase?

- Minder dan 30 minuten
- 30 tot 60 minuten
- 1 tot 2 uur
- 2 tot 6 uur
- 6 tot 12 uur
- 12 tot 24 uur
- 1 tot 2 dagen
- meer dan 2 dagen van te voren
- Overige (geef nadere toelichting)

52. Kunt u het optreden van een aanval voorspellen?

- ◯ Ja, altijd (100% van de keren)
- ◯ Ja, in 75% van de keren
- ◯ Ja, in 50% van de keren
- ◯ Ja, in 25% van de keren
- ◯ Nooit

Vragen over de aanvallen zelf

53. Heeft u ooit gezien dat uw hond zich bewust was van een aanval?

- ◯ Ja
- ◯ Nee
- ◯ Overige (geef nadere toelichting)

54. Wat doet uw hond veelal voordat een aanval optreedt?

- ◯ Hij/zij sliep
- ◯ Hij/zij werd net wakker
- ◯ Hij/zij was wakker maar lag in zijn mand/kleed te rusten
- ◯ Liep buiten
- ◯ Was aan het spelen
- ◯ Was aan het sporten
- ◯ Ik zie het nooit
- ◯ Overige (geef nadere toelichting)

55. Kunt u contact met uw hond maken tijdens de aanval?

- ◯ Ja
- ◯ Nee
- ◯ Weet ik niet

56. Indien u contact kunt maken tijdens een aanval. Hoe reageert de hond dan?

- ◯ Volledig bij, reageert gewoon
- ◯ Is anders maar lijkt wel te reageren
- ◯ Reageert totaal niet op mij

57. Hoe lang duren de aanvallen gemiddeld? Vul minuten in. Vul hier niet de tijd voor de aanval (inleidende fase) of na de aanval (post-ictale fase) in. Puur alleen de aanval.

58. Hoe lang duurde de kortste aanval?

59. Hoe lang duurde de langste aanval?

Vragen over de aanvallen zelf.

60. Hieronder staan meerdere zaken genoemd die bij een aanval kunnen optreden. Wilt u dat wat u regelmatig ziet aanklikken? Meerdere antwoorden zijn mogelijk.

- [ ] Verkrampen van de poten en de nek
- [ ] Neervallen
- [ ] Tijdens de aanval ligt de hond op een zijde
- [ ] Fiets bewegingen met de poten
- [ ] Draaien van het hoofd
- [ ] Kauwbewegingen met de bek
- [ ] Trekkingen van de aangezichtsspieren
- [ ] Urineverlies
- [ ] Ontlastingsverlies
- [ ] Speekselen
- [ ] Verwijding van de pupillen
- [ ] Rondjes lopen
- [ ] Staartjagen
- [ ] Bewustzijnsverlies
- [ ] Staren
- [ ] Contact zoeken met mensen
- [ ] Tegen meubels / andere zaken oplopen
- [ ] Ziet slecht / tijdelijk zichtverlies
- [ ] Blaffen
- [ ] Angst
- [ ] Agressie
- [ ] Overige (geef nadere toelichting)

61. Zien alle aanvallen er steeds hetzelfde uit?

- ○ Ja
- ○ Neen
- ○ Indien u neen antwoordde. Kunt u dit aangeven waarom niet?

62. is uw hond tussen de aanvallen door normaal? Dus in de periodes dat er geen aanvallen zijn?

- Ja het is verder een normale Border Collie
- Nee
- Weet ik niet
- Indien u het niet weet of nee als antwoord geeft kunt u dit dan verduidelijken?

63. Heeft u de indruk dat de linker of juist de rechterhelft van het lichaam tijdens een aanval meer actief is (denk bijvoorbeeld aan het steeds naar een zijde bewegen van het hoofd)?

- Ja er is een verschil (of het links of rechts is maakt voor dit antwoord niet uit)
- Nee ik zie geen links-rechts verschil
- Weet ik niet

64. Kunt u de aanval verkorten?

- Ja
- Neen
- Weet ik niet
- Indien ja: wat doet u dan?

Vragen over de fase na de aanval: de post-ictale fase. Deze fase kan seconden tot dagen duren.

Vaak lopen ze dan doelloos rond, zijn afwezig, zien vaak slecht en zo verder,..

65. De post-ictale fase is de fase na de aanval. Deze fase is soms niet herkenbaar, soms juist dagen aanwezig. Heeft uw hond een post-ictale fase?

- ◯ Ja
- ◯ Neen
- ◯ Weet ik niet

66. Heeft u de indruk dat uw hond zich de aanval herinnert?

- ◯ Ja
- ◯ Neen
- ◯ Weet ik niet

67. Hoe lang duurt het voordat de post-ictale fase voorbij is?

- ◯ De hond is na een aanval gelijk helemaal normaal
- ◯ Minder dan 5 minuten
- ◯ Vijf tot 15 minuten
- ◯ Vijftien tot 30 minuten
- ◯ Dertig tot 60 minuten
- ◯ Een tot twee uur
- ◯ Twee tot 6 uur
- ◯ Meer dan zes uur
- ◯ Meerdere dagen

68. Reageert de hond op u tijdens de post-ictale fase?

- ◯ Ja
- ◯ Neen
- ◯ Weet ik niet

69. Wat ziet u tijdens de post-ictale fase?

- ☐ De hond is moe
- ☐ De hond loopt doelloos rond
- ☐ De hond lijkt blind te zijn
- ☐ De hond is agressief
- ☐ De hond wil gelijk drinken
- ☐ De hond wil gelijk eten
- ☐ De hond wil wandelen
- ☐ De hond wil niets doen en blijft liggen
- ☐ De hond braakt
- ☐ De hond gaat zich uitrekken
- ☐ Overige (geef nadere toelichting)

Wat uw dierenarts zoal heeft gedaan?

70. Wat heeft uw dierenarts zoal gedaan om te komen tot de diagnose?

- [ ] Klinisch onderzoek
- [ ] Hartecho
- [ ] Bloedonderzoek
- [ ] ECG (hartfilmpje)
- [ ] ECHO hart
- [ ] EEG (Electro-encephalogram - hersenonderzoek)
- [ ] MRI scan van de hersenen
- [ ] CT scan van de hersenen
- [ ] Hersenvochtonderzoek: CSF / CSV onderzoek / liquor punctie
- [ ] Overige (geef nadere toelichting)

71. Heeft uw hond, naast de epilepsie, ook andere problemen?

- [ ] Ja
- [ ] Neen
- [ ] Indien Ja geef dan hier aan welke.

Enkele specifieke vragen over reuen en teven

72. Indien u een teefje heeft: op welke leeftijd is ze voor het eerst loops geworden?

- [ ] Ze is nog niet loops geweest
- [ ] Niet van toepassing voor mijn hond
- [ ] Haar eerste loopsheid zagen we:

73. Voor teven: is de loopsheid regelmatig?

- ( ) Ja
- ( ) Neen
- ( ) Weet ik niet

74. Voor teven: heeft uw teef ooit pups gehad?

- [ ] Ja
- [ ] Neen
- [ ] Weet ik niet
- [ ] Indien ja: hoeveel nesten?

75. Voor reuen: Heeft uw hond een normaal sexueel gedrag?

- [ ] Ja
- [ ] Neen
- [ ] Weet ik niet
- [ ] Indien neen: wat is dan abnormaal?

76. Voor reuen: heeft uw hond nakomelingen?

- [ ] Ja
- [ ] Neen
- [ ] Weet ik niet
- [ ] Indien ja: hoeveel nesten?

77.

Hoeveel aanvallen heeft uw hond de laatste maanden gehad? Tel hierbij de clusters* als een aanval.
Met een cluster wordt bedoeld: aanvallen die op elkaar volgen (binnen 24 uur) waarbij de hond tussen de aanvallen door een normaal bewustzijn heeft.

De laatste 12 maanden heeft mijn hond ... aanvallen gehad

De laatste 6 maanden heeft mijn hond ... aanvallen gehad

De laatste 3 maanden heeft mijn hond ... aanvallen gehad

De laatste maand heeft mijn hond ... aanvallen gehad

78.

Hoeveel clusteraanvallen* heeft uw hond de laatste maanden gehad?
** Met clusteraanvallen wordt bedoeld: aanvallen die op elkaar volgen (binnen 24 uur) maar waarbij de hond tussen de aanvallen door een normaal bewustzijn heeft.*

De laatste 12 maanden heeft mijn hond ... clusteraanvallen gehad

De laatste 6 maanden heeft mijn hond ... clusteraanvallen gehad

De laatste 3 maanden heeft mijn hond ... clusteraanvallen gehad

De laatste maand heeft mijn hond ... clusteraanvallen gehad

79.

Hoe vaak is er in de laatste maanden sprake geweest van een status epilepticus*?
** Status epilepticus houdt in dat de toevallen elkaar binnen zeer korte tijd opvolgen, waarbij er geen herstelfase is*

De laatste 12 maanden is er ... keer sprake geweest van een status epilepticus	
De laatste 6 maanden is er ... keer sprake geweest van een status epilepticus	
De laatste 3 maanden is er ... keer sprake geweest van een status epilepticus	
De laatste maand is er ... keer sprake geweest van een status epilepticus	

80.

De ernst van de tonisch clonische aanvallen beoordeel ik als:
(met 0 niet ernstig en 10 zeer ernstig)

Sleep de schuifregelaar naar de gewenste positie

0 (niet ernstig) 10 (zeer ernstig)

81.

Indien aanwezig: De ernst van de focale aanvallen beoordeel ik als:
(met 0 niet ernstig en 10 zeer ernstig)

Sleep de schuifregelaar naar de gewenste positie

0 (niet ernstig) 10 (zeer ernstig)

82.

Aan het gedrag van de hond kan ik voorspellen wanneer er een aanval komt; mijn hond vertoont een veranderd gedrag enige tijd voor de aanval.
(met 0 niet eens en 10 zeer eens)

Sleep de schuifregelaar naar de gewenste positie

0 (niet eens) 10 (zeer eens)

83. Hoe vaak komt uw hond op controle bij een dierenarts of specialist?

- Eens per week
- Eens per 2 weken
- Eens per 3 weken
- Eens per maand
- Eens per 2 maanden
- Eens per 3 maanden
- Eens per 4 maanden
- Eens per 5 maanden
- Eens per 6 maanden
- Eens per jaar
- Nooit
- Anders, namelijk...

84. Hoe vaak moest u in de afgelopen maanden in de avonduren of in het weekend naar een dierenarts in verband met de epilepsie van uw hond?

De afgelopen 12 maanden ... keer

De afgelopen 6 maanden ... keer

De afgelopen 3 maanden ... keer

De afgelopen maand ... keer

85. Welke medicatie gebruikt uw hond? (meerdere antwoorden mogelijk)

- [ ] Fenobarbital (merknamen: Phenoral, Phenoleptil en Soliphen)
- [ ] Fenytoine (Epitard)
- [ ] Imepitoine (Pexion)
- [ ] Kaliumbromide (merknamen: Epikal, Libromide)
- [ ] Neusspray midazolam
- [ ] Diazepam, valium, stesolid
- [ ] Gebapentine (merknamen: Neurontin of Gabapentine)
- [ ] Levetiracitam (merknamen: Keppra of levetiracetam)
- [ ] Geen medicatie
- [ ] Anders, namelijk...

86.

Welke neveneffecten van de medicatie ziet u bij uw hond?
(er zijn meerdere antwoorden mogelijk)

- [ ] Sloomheid
- [ ] Meer slapen
- [ ] Rusteloosheid
- [ ] Prikkelbaar
- [ ] Wankel zijn/ ongecoördineerd
- [ ] Spierzwakte / zwakte
- [ ] Toename eetlust
- [ ] Gewichtstoename
- [ ] Braken
- [ ] Diarree
- [ ] In huis poepen
- [ ] Meer drinken
- [ ] Meer plassen
- [ ] Hoesten
- [ ] Huiduitslag
- [ ] GEEN. Mijn hond heeft geen bijwerkingen.
- [ ] Anders, namelijk...

87. Maakt uw hond gebruik van alternatieve behandelingsmethoden?

- ○ Ja
- ○ Nee

88.

Indien van toepassing: Van welke alternatieve behandelingsmethode(n) maakt uw hond gebruik? (meerdere antwoorden mogelijk)

- ☐ Fytotherapie
- ☐ CBD olie
- ☐ CBD/THC olie
- ☐ MCT olie
- ☐ Speciaal dieet
- ☐ Muziektherapie
- ☐ Geen enkele. Ik geef geen alternatieve behandeling
- ☐ Anders, namelijk...

89.

Terugkijkend naar de periode voordat uw hond epilepsie kreeg, als die periode 100% goed was. Hoe scoort u uw hond dan nu? Het cijfer 0 drukt uit dat u de situatie erg slecht vindt en 100 betekent dat het erg goed gaat.

Sleep de schuifregelaar naar de gewenste positie

0 het gaat erg slecht — 100 het gaat erg goed

90.

In de laatste 3 maanden heb ik mij zorgen gemaakt over de frequentie van de aanvallen bij mijn hond.

Sleep de schuifregelaar naar de gewenste positie. Met 0 geen zorgen en 10 erg veel zorgen.

0 geen zorgen — 10 erg veel zorgen

91.

De ernst van de aanvallen bij mijn hond is acceptabel voor mij.

Sleep de schuifregelaar naar de gewenste positie. Met 1 het gaat erg goed en 10 het gaat erg slecht

0 het gaat erg goed — 10 het gaat erg slecht

92.

Ik durf mijn hond alleen thuis te laten.

Sleep de schuifregelaar naar de gewenste positie. Met 1 geen enkel probleem en 10 ik durf hem/haar niet alleen te alten.

1 geen enkel probleem — 10 ik durf hem/haar niet alleen te laten

93.

Het zorgen voor mijn hond met epilepsie beperkt mij in mijn dagelijkse bezigheden; het zorgt voor een afname van mijn eigen kwaliteit van leven.

Sleep de schuifregelaar naar de gewenste positie. Met 1 niet eens en 10 zeer eens.

1 niet eens — 10 zeer eens

94.

Het zorgen voor mijn hond met epilepsie is het waard.

Sleep de schuifregelaar naar de gewenste positie. Met 1 niet eens en 10 zeer eens.

1 niet eens — 10 zeer eens

95.

Het toedienen van de medicatie bij mijn hond levert problemen op.

Sleep de schuifregelaar naar de gewenste positie. Met 1 niet eens en 10 zeer eens.

1 niet eens 10 zeer eens

96.

De neven-effecten van de medicatie bij mijn hond zijn acceptabel voor mij.

Sleep de schuifregelaar naar de gewenste positie. Met 1 geen probleem en 10 veel bijwerkingen: niet acceptabel.

1 geen probleem 10 veel bijwerkingen: niet acceptabel.

97.

De kosten van de behandeling van epilepsie zijn acceptabel voor mij.

Sleep de schuifregelaar naar de gewenste positie. Met 1 niet acceptabel en 10 zeer acceptabel

1 niet acceptabel 10 zeer acceptabel

98.

Is het voor u een probleem om bij de dierenarts of specialist op consult te gaan (voor bijvoorbeeld controles, onderzoek van de bloed concentraties van de medicatie)?

Sleep de schuifregelaar naar de gewenste positie. Met 1 niet eens en 10 zeer eens.

1 niet eens 10 zeer eens

99.

Scoor de kwaliteit van leven van uw hond.

Sleep de schuifregelaar naar de gewenste positie. Met 1 zeer slecht en 10 uitstekend.

1 zeer slecht | 10 uitstekend

100. Welke fase van epilepsie vindt u de grootste aanslag op de kwaliteit van leven van uw hond?

- Inleidende fase / prodromale fase
  Dit is een fase van abnormaal gedrag, mogelijk gepaard gaande met braken; het duurt enkele seconden tot dagen.
- De aanval / ictus
  Dit is een fase van bewustzijnsverlies en krampen; dit kan gepaard gaan met blaffen, kwijlen, plassen en poepen; dit duurt seconden tot minuten.
- De herstelfase / post-ictale fase
  Dit is een fase waarin de hond soms niet kan lopen, slaperig is, onrustig en mogelijk agressief is; het duurt seconden tot weken.
- Geen specifieke fase

101. Helaas is het erg moeilijk om volledige aanvalsvrijheid te verkrijgen. Maar wat vindt u redelijkerwijs acceptabel?

- Een aanval per week
- Een aanval per 2 weken
- Een aanval per 3 weken
- Een aanval per maand
- Een aanval per 2 maanden
- Een aanval per 3 maanden
- Een aanval per 4 maanden
- Een aanval per 5 maanden
- Een aanval per 6 maanden
- Een aanval per jaar
- Vrij van aanvallen

102. Hartelijk bedankt voor uw medewerking aan dit onderzoek. Indien u interesse heeft in de resultaten van het onderzoek, dan kunt u hieronder uw e-mailadres doorgeven, dan zult u de uiteindelijke onderzoeksresultaten ontvangen.
Indien u vragen en/of opmerkingen heeft, kunt u die hier plaatsen.
